# Development and validation of the pandemic fear perception and adaptability scale

**DOI:** 10.3389/fpsyg.2025.1661940

**Published:** 2025-10-14

**Authors:** Mu Wang, Haiyan Qi, Yanyan Chen, Mian Li

**Affiliations:** ^1^Interdisciplinary Sciences Institute, Hefei University of Technology, Hefei, Anhui, China; ^2^Anhui Women’s Compulsory Drug Rehabilitation Center, Hefei, Anhui, China; ^3^Institute of Intelligent Machines, Hefei Institute of Physical Science, Chinese Academy of Sciences, Hefei, Anhui, China; ^4^School of Management, Hefei University of Technology, Hefei, Anhui, China

**Keywords:** fear, psychology, pandemic, scale, perception

## Abstract

**Introduction:**

Individuals exhibit specific behavioral responses to fear and stress. The Pandemic Fear Perception and Adaptability Scale (PFPA) is a novel instrument designed to assess fear perception and behavioral adaptability in the context of pandemics.

**Methods:**

A cross-sectional survey was conducted with 923 participants in China to evaluate the psychometric properties of the PFPA. After expert review, a 7-item scale was developed, comprising three subscales: self-efficacy, perceived susceptibility, and perceived threat. Classical test theory, confirmatory factor analysis (CFA) and Rasch modeling were used to assess the scale’s reliability and validity.

**Results:**

The PFPA demonstrated good reliability, with an internal consistency (Cronbach’s *α* = 0.700) and test-retest reliability (ICC(2,1) = 0.781, *p* < 0.001). Evidence of validity was supported by an average variance extracted of 0.563 and a composite reliability of 0.898. CFA indicated excellent model fit (χ²(11) =15.123, *p* = 0.177; CFI = 0.998, GFI = 0.995, TLI = 0.995, RMSEA = 0.020). Differential item functioning analysis showed minimal bias across gender and age.

**Discussion:**

This study establishes the PFPA as a reliable and valid tool for assessing fear perception and behavioral adaptability, with potential to evaluate these constructs in other pandemic contexts. Given its initial development in a Chinese context, future research should examine its applicability across diverse cultural and linguistic settings.

## 1 Introduction

In recent years, large-scale infectious disease outbreaks, such as COVID-19, have highlighted the urgent need to understand not only biomedical responses, but also the psychological and behavioral dynamics of affected populations. While previous studies have often emphasized emotional symptoms or clinical disorders, a more comprehensive framework requires linking cognitive appraisals of risk with behavioral adaptation.

Fear among the general public is one of the main psychological responses caused by pandemics (Mertens et al., 2023). The fear of infection during an epidemic not only leads to mental disorders and worsens existing mental health issues, but also alters individual behaviors (Colizzi et al., 2020; Mertens et al., 2024). According to the Transactional Theory of Stress and Coping (Biggs et al., 2017), stress and fear arise not directly from external events, but from individuals’ cognitive assessment of these events. Individuals regulate fear either by managing emotions or by engaging in problem-solving actions, which subsequently shape their behavioral responses during crises. The choice of coping strategies will influence the individual’s ultimate response to fear. When facing an inescapable regional crisis such as an epidemic, individuals also take corresponding actions to regulate or soothe their emotions. Such as keeping social distance, wearing masks and other engaging in stockpiling behaviors (Huterska et al., 2021; Rayburn et al., 2022). These fear-induced behaviors typically arise spontaneously from individuals seeking to alleviate their perceived fear, rather than being compelled (Harper et al., 2021; Wise et al., 2020).

Beyond this general model, two established theories are particularly relevant to the present study. The Health Belief Model (HBM) posits that perceived susceptibility, perceived severity (threat), and self-efficacy jointly shape preventive behaviors during health crises (Green et al., 2020). Similarly, Protection Motivation Theory (PMT) emphasizes the interplay of threat appraisal (susceptibility, severity) and coping appraisal (self-efficacy, response efficacy) in driving protective actions (Marikyan and Papagiannidis, 2023). Together, these frameworks highlight that pandemic-related fear is not a purely emotional reaction, but a multidimensional construct grounded on cognitive evaluations of risk and coping ability.

Self-efficacy refers to one’s belief in the capacity to successfully perform specific tasks or manage particular situations. It promotes goal-setting, sustained effort, and recovery from setbacks (Schwarzer and Luszczynska, 2008). Importantly, individuals can develop self-efficacy that enables them to engage in protective behaviors to counteract fear (Zlomuzica et al., 2015). Fear is also positively correlated with perceived susceptibility (Kim and Chang, 2020; Yıldırım et al., 2021), which is how someone views their own vulnerability to a specific threat or health concern. Perceived susceptibility describes the subjective assessment of personal vulnerability to a specific threat. Perceived susceptibility is linked to health risk perception and risk avoidance, such as taking vaccines or wearing masks in COVID-19 outbreak (Bin et al., 2024). Higher perceived susceptibility has consistently been linked to greater health risk perception and preventive actions, such as vaccination uptake and mask-wearing (Vogel et al., 2021; Weinstein et al., 1991). Moreover, perceived threat refers to the evaluation of the potential harm posed by a disease or health risk. It is often considered a precursor to fear, acting as its immediate trigger (Mandik, 2022). Finally, the interaction among these three constructs has been observed in multiple epidemics. Self-efficacy, perceived susceptibility, and perceived threat have been widely observed across various epidemics, causing fear and influencing individual behaviors (Mo et al., 2021; Zhang et al., 2022). Individuals with higher self-efficacy, lower perceived susceptibility, and lower perceived threat may exhibit greater adaptability and resilience when confronting fear-inducing situations, while those with opposite perceptions may experience heightened fear and may struggle to adapt effectively (De Zwart et al., 2009; Zhang et al., 2014), which has been found in public health emergencies (Zhao et al., 2023). Taken together, self-efficacy, perceived susceptibility, and perceived threat play crucial roles in shaping individuals’ perception of fear, while also influencing their behaviors and the perception of risk in pandemic may not be the same for everyone (Filindassi et al., 2022). Furthermore, recent evidence suggests that self-efficacy and risk perceptions play distinct roles in predicting protective behaviors such as social distancing and mask-wearing in pandemic, underscoring their role in promoting psychological resilience (Caprara et al., 2024; Duong et al., 2024).

Building on these frameworks, we argue that pandemic-related fear should not be understood solely as an emotional state, but as a multidimensional construct encompassing perceptions of susceptibility, threat, and coping ability. This perspective provides a strong theoretical basis for the development of the Pandemic Fear Perception and Adaptability Scale (PFPA), which is designed to capture these interrelated components. Importantly, the PFPA does not only capture cognitive perceptions of pandemic-related fear, but also aims to assess the individual’s *adaptability* in the face of such fear. In this context, adaptability refers to the capacity to regulate fear through self-efficacy and to translate perceived susceptibility and threat into constructive protective behaviors rather than maladaptive responses. Accordingly, we hypothesized that: (H1) Consistent with the Health Belief Model and Protection Motivation Theory, the PFPA will demonstrate a three-factor structure corresponding to self-efficacy, perceived susceptibility, and perceived threat; and (H2) Each subscale will exhibit satisfactory psychometric properties, including internal consistency and validity indicators, while acknowledging potential limitations for the brief two-item self-efficacy subscale.

Previous studies have developed several scales to assess fear, including the Fear Survey Schedule (FSS-III), the Fear Questionnaire (FQ) (Arrindell and Emmelkamp, 1984), the Fear of COVID-19 Scale (FCV-19S) (Ahorsu et al., 2020) and the Coronavirus Anxiety Scale (Jovanović et al., 2024). These instruments have provided valuable insights into fear intensity, and the CAS in particular has demonstrated strong psychometric robustness in both general and clinical populations. However, they are largely limited to emotional or symptomatic dimensions of fear. They do not explicitly integrate the cognitive constructs outlined in HBM and PMT, such as self-efficacy, susceptibility, and threat perception, nor do they systematically link these constructs with adaptive or maladaptive behaviors. Thus, there remains a conceptual and methodological gap in measuring pandemic fear as a multidimensional phenomenon grounded in established health psychology theories. The PFPA is designed to address this gap by integrating these theoretical perspectives into a concise psychometric instrument. A real-time assessment of public fear, its cognitive underpinnings, and the resultant behavioral changes is crucial for the development of timely and effective policies to mitigate fear and guide the population through epidemic situations.

The PFPA was developed and tested during the immediate aftermath of China’s sudden lifting of COVID-19 restrictions in December 2022. At that time, the rapid spread of infection and widespread public anxiety created an exceptional psychological context. Data collected under these circumstances are uniquely valuable, as they capture fear perceptions and adaptive responses in a rare moment of collective uncertainty. This dataset captures the intensity of fear during China’s COVID-19 policy shift, offering insights into public reactions to sudden health crises. While the urgency of the situation constrained some methodological choices, this context also provides an unparalleled opportunity to study fear and adaptability under real-world crisis conditions.

## 2 Methods

### 2.1 Consent to participate

This study developed and validated a novel scale to assess individuals’ fear perception and adaptability in pandemic. It was approved by Ethics Committee of Institute of Intelligent Machines, Chinese Academy of Sciences, Hefei. The questionnaire (including the scale) was published online. All procedures performed in this study involving human participants were in accordance with the ethical standards of the institutional research committee and with the 1964 Helsinki Declaration and its later amendments or comparable ethical standards. The online survey was restricted to adults aged 18 years or older. Before beginning the questionnaire, participants were presented with an information page describing the study purpose, procedures, and data protection. Proceeding to complete the survey indicated their consent. No identifying information (e.g., names, phone numbers, ID numbers) was collected, ensuring complete anonymity.

### 2.2 Development of the scale

Firstly, we conducted a comprehensive review of fear-related scales and behaviors that fear may induce during epidemics. We organized an initial pool of 18 items based on existing fear scales and other psychological measures, focusing on three aspects: self-efficacy, perceived susceptibility, and perceived threat. Additionally, we identified four of the most common behavioral changes from academic articles, social media, official news, and field observations as external validators. To evaluate content validity, we invited a panel of eight experts, including two behavioral scientists, two clinical psychologists, two nurses specializing in infectious disease care, and two clinical physicians. The experts were asked to independently assess each item on several criteria: (a) relevance to the construct, (b) clarity of wording, (c) theoretical representativeness, and (d) practical comprehensibility for the general population. Ratings were made on a 4-point scale (1 = not relevant, 4 = highly relevant). There were three iterative rounds of review, each including at least six of the experts, with partial changes in panel composition to avoid groupthink. Finally, 11 of the original 18 items were removed due to low relevance or redundancy, and the four behavioral external validators were refined into three. The final version of the Pandemic Fear Perception and Adaptability Scale (PFPA) contained seven items: two items assessing self-efficacy, three assessing perceived susceptibility, and two assessing perceived threat. All items are rated on a 7-point Likert scale (1 = totally disagree, 7 = strongly agree).

The seven items of PFPA are shown below:

Subscale ‘Self-efficacy’ included:

1. ‘I believe I am able to perform the protective behaviors mentioned above to prevent or cope with the pandemic (COVID-19).’
2. ‘Doing the above actions can help avoid the pandemic (COVID-19) or recover better if I contract the pandemic (COVID-19).’

Subscale “Perceived susceptibility” included:

1. ‘I am at risk of getting infected by the pandemic (COVID-19).’
2. ‘I might get infected by the pandemic (COVID-19).’
3. ‘I might have been infected with COVID-19.’

Subscale ‘Perceived Threat’ included:

1. ‘The pandemic (COVID-19) is very harmful.’
2. ‘The pandemic (COVID-19) is a serious threat to us.’

The two self-efficacy items were explicitly anchored to the three external validators. Specifically, participants were first asked whether they had engaged in these behaviors (binary yes = 1/no = 2). Then, the self-efficacy subscale assessed their perceived ability to perform these same behaviors and the belief that performing them would be effective. This design ensured that self-efficacy ratings were grounded in concrete, context-specific behaviors rather than abstract general beliefs. These questions are not in the PFPA, but as the external behavioral validators.

Fear-induced behavior-change questions included:

1. ‘Have you tried to avoid going out and other social activities due to the pandemic (COVID-19)?’
2. ‘Have you attempted to purchase and stock up on medications or other items related to preventing or treating the pandemic (COVID-19)?’
3. ‘Do you always wear medical masks when going out, even N95 masks? Have you increased the frequency and duration of handwashing?’

At last, 8 individuals (5 men and 3 women, mean age = 27.925 years, SD = 5.233) were asked to answer the initial scale and a four-point Likert scale (do not understand, partial understand, understand and totally understand) that was utilized to assess whether they understood the meaning of each item. Seven individuals indicated ‘totally understand’ and one expressed ‘understand’. The total score of PFPA ranges from 7 to 49. Higher score of overall PFPA represents more severe fear of the pandemic. The higher score in subscales indicates higher self-efficacy, more perceived susceptibility and more threat individuals felt.

In addition to the PFPA, we included six affect-related items adapted from the State–Trait Anxiety Inventory (STAI). These items assessed feelings of calmness, security, peacefulness, happiness, and relaxation, as well as one item reflecting fearfulness. Most items were positively worded, representing the conceptual opposite of fear. Responses were recorded on a 4-point Likert scale (1 = totally disagree, 4 = strongly agree). Although these items do not constitute a complete STAI subscale, they were included to provide preliminary convergent validity evidence. The detailed information for scales of PFPA, external validators and STAID items can be checked in Supplementary Material.

### 2.3 Participants

The questionnaire was distributed online via a survey platform called ‘Questionnaire Star’, which is a professional online platform for surveys, exams, assessments, and voting, widely utilized in commercial, research, and personal interest fields in China. The questionnaires were distributed and collected at the end of December 2022 and the beginning of January 2023, during which period of time the local government lifted most of the COVID-19 prevention and control measures. Participation in this study was entirely voluntary.

**Figure 1:**
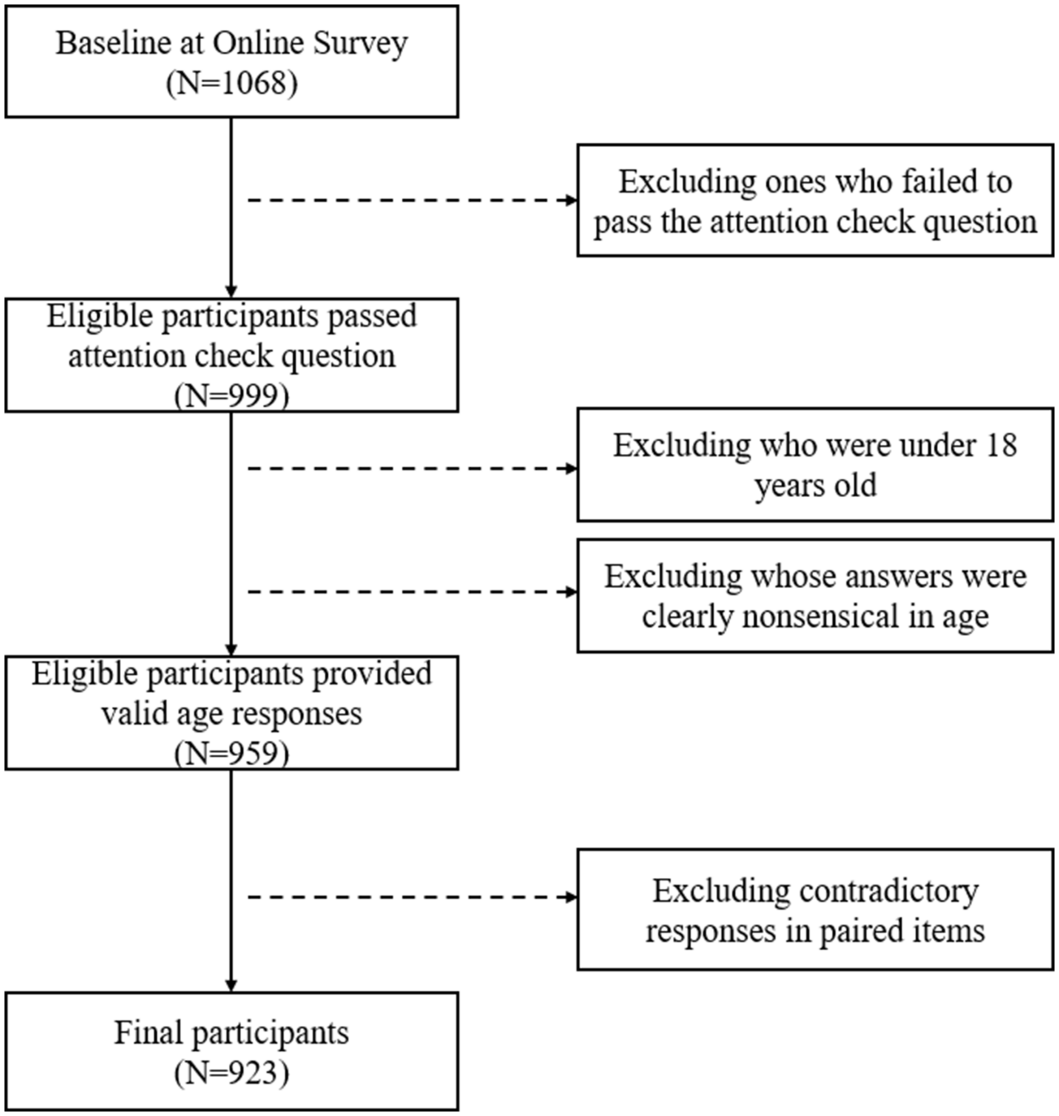
Flowchart of eligible participants screening.

A total of 1,068 individuals completed the online survey. To ensure data quality and ethical compliance, a multi-step screening procedure was applied to the raw responses. First, an attention check item was embedded in the questionnaire (“Please select ‘Moderate’ for this question”). Participants who failed this check were excluded, resulting in 999 valid participants. Second, individuals younger than 18 years old, as well as respondents whose answers were clearly nonsensical (e.g.: not a number), were removed, leaving 959 participants. Third, two pairs of questions with contradictory meanings were used to further detect random or careless responses, ‘I feel very secure’ and ‘I feel very fearful’. The answer of ‘I feel very fearful’ did reverse-scoring first. Participants who endorsed mutually exclusive extreme values (e.g., responding “1” to one item and “4” to its opposite) were excluded. After this final screening step, the analytic sample comprised 923 participants. The flowchart of participants screening is shown in Figure 1. Thirty participants were asked to answer the scale again the day after first participating, for the purpose of test–retest. All participants were Chinese and speaking Mandarin. The background information of participants is shown in Table 1.

**Table 1:** Participants’ demographic information.

Demographic characteristics	Mean (SD)
Age	29.026(11.877)
Sex	
Men	*n* = 561(60.780%)
Women	*n* = 362(39.220%)
Have you ever been infected with COVID-19	
Yes	598(64.789%)
No	198(21.452%)
Currently infected	127(13.759%)
Current living situation	
Living alone	245(26.544%)
Living with friends	128(13.868%)
Living with family	550(59.588%)
Main ways to know information about COVID-19	
Official news (from governments or medical institutions)	344(37.270%)
Social media	454(49.187%)
From friends	95(10.293%)
I have basic knowledge of the virus myself (such as medical workers)	15(1.625%)
Others	15(1.625%)
Commuting ways	
Commuting	271(29.361%)
Work from home	157(17.010%)
Freelance	183(19.827%)
Students	250(27.086%)
Others	62(6.717%)

### 2.4 Statistical analysis

The psychometric evaluation of the PFPA was conducted using both Classical Test Theory (CTT) and the Rasch model. CTT analyses included internal consistency (Cronbach’s *α*), test–retest reliability, intraclass correlation coefficients, corrected item–total correlations, mean inter-item correlations, and the standard error of measurement (SEM).

Construct validity was assessed within a Confirmatory Factor Analysis (CFA) framework using Structural Equation Modeling (SEM). The CFA was performed on the seven Likert items, testing the hypothesized three-factor model (self-efficacy, perceived susceptibility, perceived threat). Model fit was evaluated using χ^2^/df, Comparative Fit Index (CFI), Tucker–Lewis Index (TLI), Goodness of Fit Index (GFI), and Root Mean Square Error of Approximation (RMSEA). In addition, Composite Reliability (CR) and Average Variance Extracted (AVE) were computed to assess convergent validity. Standardized factor loadings were also reported. CFA was implemented, with the Maximum Likelihood estimator, chosen given the sample size and approximate multivariate normality assumptions, which were tested prior to analysis.

Rasch analysis included item and person separation reliability, item and person separation indices, point–measure correlations (PT-Measure), differential item functioning (DIF) across sex and age (median split at 25 years), and infit and outfit mean-square statistics (MNSQ).

For external validity, Pearson or Spearman correlations (as appropriate) were computed between PFPA scores and (a) six STAI-derived items (positively worded affective states) and (b) three binary behavioral validators.

All tests were two-tailed with a significance threshold of *p* < 0.05, and 95% confidence intervals were reported where applicable (90% intervals were used for RMSEA as per standard SEM practice). Results are presented to three decimal places (except Rasch outputs, which were limited by WINSTEPS formatting). Analyses were conducted in SPSS 25.0, including function AMOS, and WINSTEPS 3.75.0.

## 3 Results

There were 923 participants, aged from 18 to 83 years old, average aged 29.026 years old (SD ± 11.877), finished the whole questionnaire, including the demographic information and the scale (PFPA). Of 923 individuals, 561 were men and 362 were women. Five hundred and ninety-eight participants had experienced infecting COVID-19, 198 had not, and 127 were in the process of recovering from COVID-19.

### 3.1 Classical test theory**—**reliability

Classical Test Theory (CTT) analysis was conducted to evaluate the reliability of the PFPA scale with a sample of 923 participants. As shown in Table 2, the overall scale demonstrated acceptable internal consistency (Cronbach’s *α* = 0.700), with subscale alphas: 0.703 (Self-efficacy), 0.688 (perceived susceptibility), and 0.833 (perceived threat). Test–retest reliability was evaluated using the Intraclass Correlation Coefficient (ICC(2,1), two-way random effects, single measure, absolute agreement) for a subsample of 30 participants, with the 95% confidence interval calculated using Fisher’s Z transformation [0.590, 0.891], *p* < 0.001. The common inter-item correlation was 0.255, indicating moderate item interrelatedness, and the SEM was 3.936, reflecting adequate precision for a new scale. Item-level statistics, including corrected item-total correlations (0.205–0.528) and alpha if item deleted (0.638–0.714), confirmed no item substantially reduced reliability, as shown in Table 3.

**Table 2:** Scale validation assessment from Classical Test Theory and Rasch model.

Analysis methods	Value	Suggested cutoff
Classical test theory- Reliability (*N* = 923)		
Internal consistency (Cronbach’s alpha)		
Overall scale	0.700	> 0.7
Self-efficacy	0.703	> 0.7
Perceived susceptibility	0.688	> 0.7
Perceived threat	0.833	> 0.7
Test–retest (*n* = 30)	0.781*	>0.7
Common inter-item correlation	0.255	≈0.15 ~ 0.50^a^
Standard error of measurement	3.936	the smaller, the better
Classical test theory- Validity		
Composite reliability		
Overall scale	0.898	> 0.7
Self-efficacy	0.730	> 0.7
Perceived susceptibility	0.717	> 0.7
Perceived threat	0.835	> 0.7
Rasch model (*N* = 923)		
Model standard error		the smaller, the better
Item separation reliability from Rasch	0.97	> 0.7
Item separation index from Rasch	5.83	> 2
Person separation reliability from Rasch	0.61	> 0.7
Person separation index from Rasch	1.25	> 2
Confirmatory factor analysis (*N* = 923)		
Chi-square minimum discrepancy	15.123	the smaller, the better
*p*-value	0.177	>0.05
Chi-square minimum discrepancy/DF	1.375	<2
Comparative fit index	0.998	>0.900
Goodness of fit index	0.995	>0.900
Tucker-Lewis index	0.995	>0.900
Root mean square error of approximation	0.020	<0.080
Average variance extracted	0.563	>0.50

*p < 0.05. ^a^Values between 0.15 and 0.50 indicate moderate inter-item correlations, ensuring items measure the same construct without redundancy.

**Table 3:** Means and SDs, factor loadings, item total correlations and item properties.

Items	Mean ± SD	Corrected item-total correlation	Cronbach’s alpha if item deleted	Standardized loading	*R* ^2^	Infit mean square	Outfit mean square	PT-Measure (Correction)	DIF contrast across gender ^a b^	DIF contrast across age ^a c^
Item 1	5.858 ± 1.267	0.294	0.692	0.854	0.730	1.16	1.36	0.43 (0.44)	0.06	−0.32
Item 2	5.232 ± 1.594	0.205	0.714	0.651	0.424	0.90	0.88	0.45 (0.52)	−0.07	−0.30
Item 3	4.926 ± 1.83	0.492	0.642	0.703	0.494	0.96	1.11	0.57 (0.56)	0.00	0.21
Item 4	5.439 ± 1.700	0.489	0.645	0.748	0.559	0.93	1.01	0.52 (0.49)	−0.05	0.19
Item 5	5.202 ± 2.188	0.381	0.682	0.543	0.295	0.82	0.82	0.48 (0.52)	0.00	0.16
Item 6	5.484 ± 1.555	0.528	0.638	0.851	0.724	0.83	0.86	0.54 (0.49)	0.06	0.00
Item 7	5.507 ± 1.550	0.523	0.640	0.839	0.703	1.07	1.03	0.53 (0.49)	0.00	0.00

^a^ DIF contrast > 0.5 indicates substantial DIF. ^b^ DIF contrast across gender for men and women. ^c^ DIF contrast across age, using median age (25) as dividing point.

**Figure 2:**
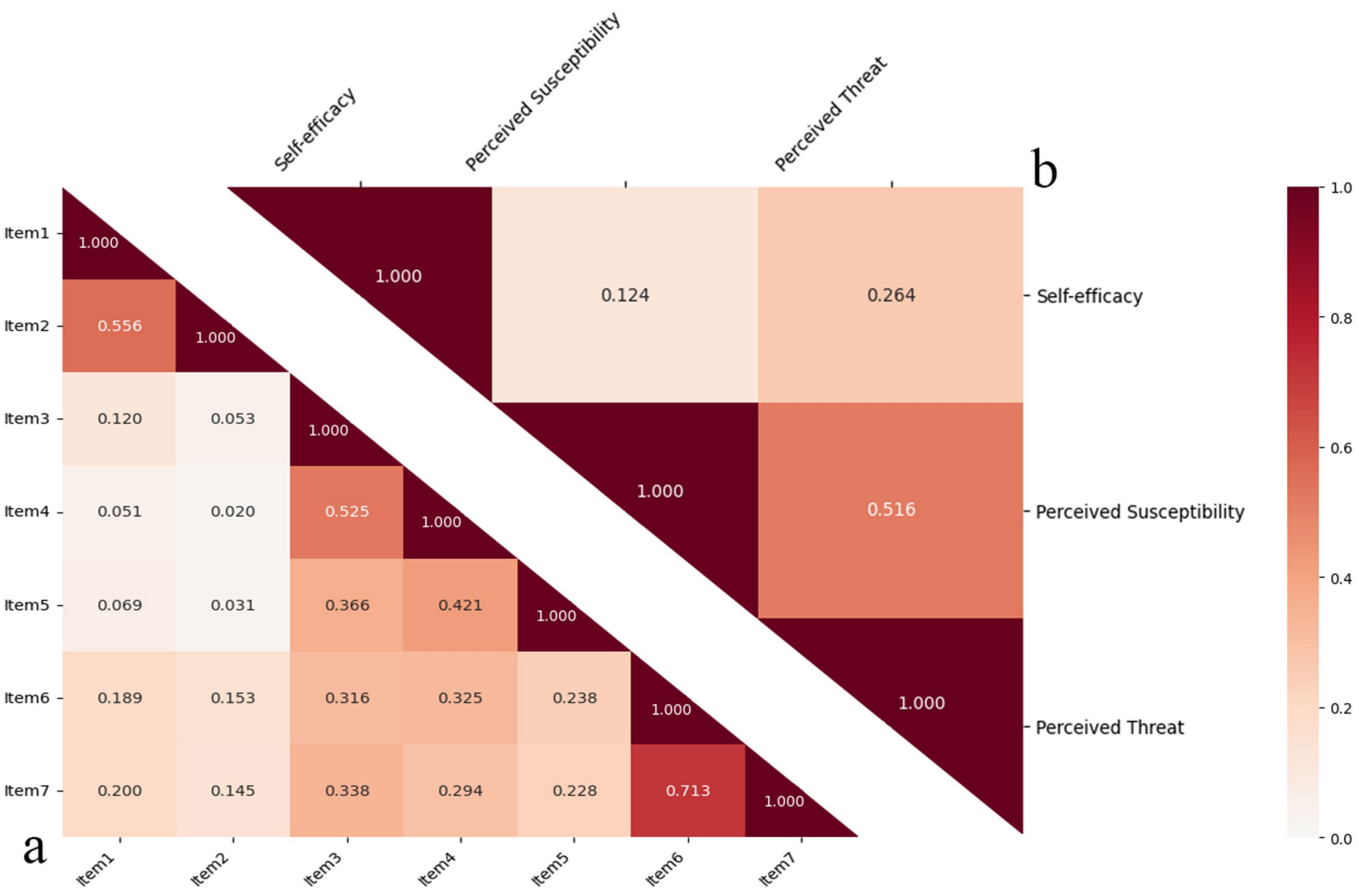
Heatmaps of inter-item and subscale correlations for PFPA scale. **(a)** Illustrates the correlation between items and **(b)** demonstrates the correlation between subscales. The numbers in the chart represent the correlation coefficient. The darker (redder) the color, the stronger the correlation.

As shown in Figure 2, inter-item and subscale correlations were examined to assess the internal consistency of the PFPA scale (*N* = 923). The average inter-item correlation was 0.255, indicating moderate item interrelatedness suitable for a multidimensional scale. Item correlations ranged from 0.020 (Item2-Item4) to 0.713 (Item6-Item7), with stronger correlations within subscales (e.g., Item3-Item4, *r* = 0.525; Item6-Item7, *r* = 0.713) supporting their coherence. Subscale correlations ranged from 0.124 (Self-efficacy and Perceived Susceptibility) to 0.516 (Perceived Susceptibility and Perceived Threat), confirming related but distinct constructs. These findings, visualized in heatmaps (Figure 2), reinforce the scale’s reliability while supporting discriminant validity, as subscale correlations remained below 0.85.

### 3.2 Classical test theory**—**validity

As shown in Table 2, Confirmatory Factor Analysis (CFA) was conducted to evaluate the construct validity of the Pandemic Fear Perception and Adaptability Scale (PFPA) with a sample of 923 participants. As shown in Table 2, the overall scale CR was 0.898, with subscale CRs of 0.730 (self-efficacy), 0.717 (perceived susceptibility), and 0.835 (perceived threat), all exceeding the 0.7 threshold for acceptable reliability.

Moreover, the hypothesized three-factor model (self-efficacy, perceived susceptibility, perceived threat) demonstrated excellent fit with the data: χ^2^(11) = 15.123, *p* = 0.177; χ^2^/df = 1.375; CFI = 0.998; GFI = 0.995; TLI = 0.995; RMSEA = 0.020 (90% CI: 0.000–0.043, PCLOSE = 0.989); RMR = 0.046; AGFI = 0.988; AVE = 0.563. The non-significant χ^2^ value indicates no substantial deviation between the observed and hypothesized covariance matrices, supporting model fit. The χ^2^/df ratio of 1.375, well below the threshold of 2.0, suggests a parsimonious model. CFI, GFI, and TLI values exceeding 0.95, along with an RMSEA of 0.020 and low RMR of 0.046, further confirm excellent fit.

### 3.3 Rasch model

**Figure 3:**
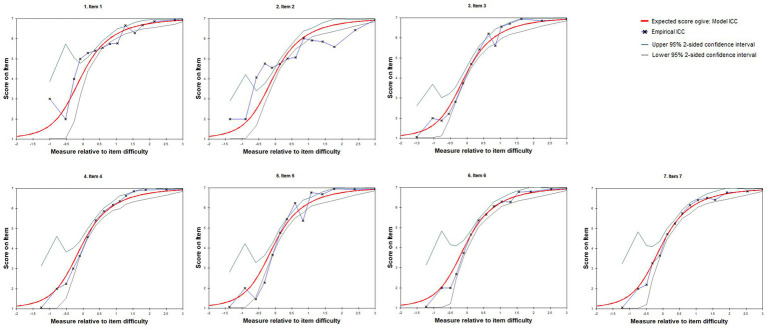
Item characteristic curves (ICCs) for PFPA scale items. ICCs illustrate the expected item scores (vertical axis, 1–7 Likert scale) as a function of the latent trait relative to item difficulty (horizontal axis, logits). Empirical points represent observed response probabilities, with upper and lower 95% confidence intervals indicating model fit. The plot evaluates item performance and response category appropriateness in the Rasch model.

Rasch analysis was performed on 923 participants. As shown in Table 2, the item separation reliability was 0.97, and the item separation index was 5.83, indicating excellent item differentiation. The person separation reliability was 0.61, with a person separation index of 1.25, acceptable for early-stage development. As shown in Table 3, Infit and Outfit Mean Square (MNSQ) values ranged from 0.82 to 1.16 (Infit) and 0.82 to 1.36 (Outfit), within the acceptable range of 0.5–1.5. ICC plots (Figure 3) showed good alignment for most items, with Items 2 and 5 exhibiting empirical points exceeding 95% confidence intervals in several categories, indicating slight misfit possibly due to response variability. Differential item functioning (DIF) analysis revealed minimal bias across gender (|DIF| ≤ 0.06) and age (|DIF| ≤ 0.21), supporting measurement invariance.

### 3.4 Convergent validity with external and behavioral measures

Convergent validity of the PFPA scale was assessed by correlating its total and subscale scores with six affect-related items adapted from the STAID and a behavioral total score using Pearson correlations (*N* = 923). The Cronbach’s Alpha of STAID itself is 0.866. The PFPA total score showed a significant negative correlation with the STAI-derived items (*r* = −0.204, 95% CI [−0.265, −0.141], *p* < 0.001), as well as its subscales: self-efficacy (*r* = 0.227, 95% CI [0.165, 0.287], *p* < 0.001), perceived susceptibility (*r* = −0.305, 95% CI [−0.362, −0.245], *p* < 0.001), and perceived threat (*r* = −0.215, 95% CI [−0.276, −0.153], *p* < 0.001).

For the external behavioral validators, the PFPA total score was again significantly correlated (*r* = −0.219, 95% CI [−0.280, −0.157], *p* < 0.001), as were its subscales: self-efficacy (*r* = −0.155, 95% CI [−0.217, −0.091], *p* < 0.001), perceived susceptibility (*r* = −0.148, 95% CI [−0.211, −0.084], *p* < 0.001), and perceived threat (*r* = −0.173, 95% CI [−0.235, −0.110], *p* < 0.001).

## 4 Discussion

This study introduced the Pandemic Fear Perception and Adaptability Scale (PFPA), a novel tool to assess individuals’ pandemic-related fear and adaptability. The PFPA demonstrated satisfactory reliability and validity across multiple psychometric approaches (CTT, Rasch model, CFA), confirming its theoretical foundation in the Health Belief Model and Protection Motivation Theory. The three-factor structure, including self-efficacy, perceived susceptibility, and perceived threat, captures the multidimensional nature of pandemic fear and distinguishes cognitive appraisals from emotional reactivity. This approach moves beyond existing fear measures such as the FCV-19S (Ahorsu et al., 2020), which primarily emphasizes emotional symptoms, and the CVS (Lieven, 2023), which assesses physiological and anxiety-related responses. By integrating appraisal-based constructs with adaptability, the PFPA offers a complementary framework to these scales.

Our findings also resonate with previous psychometric work on pandemic fear, which consistently highlights the interaction between cognitive perceptions and behavioral adaptation (Cummings et al., 2022; Pakpour et al., 2021). Importantly, PFPA’s inclusion of self-efficacy provides a practical lens to understand how beliefs about coping capacity influence protective behavior, a factor underexplored in other pandemic fear instruments. This is particularly relevant given evidence that self-efficacy strongly predicts adherence to preventive measures such as mask wearing, hand hygiene, and social distancing (Scholz and Freund, 2021; Wongrith et al., 2024).

The cross-sectional correlations between PFPA subscales and behavior indices in our study further illustrate this linkage. Higher perceived threat and susceptibility were moderately associated with reported preventive actions, while self-efficacy contributed to confidence in sustaining these behaviors. These findings underscore the PFPA’s potential utility in identifying individuals or groups at risk of maladaptive responses (e.g., excessive avoidance or panic buying) versus adaptive protective behaviors.

The present study also revealed meaningful associations between PFPA subscales, positive affect, and external behavioral validators. As expected, higher levels of perceived susceptibility and perceived threat were negatively correlated with positive affect items derived from the STAID, reflecting the emotional burden of fear. In contrast, self-efficacy showed a positive correlation with positive affect, suggesting that individuals who believe in their capacity to prevent or cope with pandemic threats are more likely to experience calmness, security, and relaxation. This finding is consistent with Bandura’s social cognitive theory, which emphasizes that self-efficacy functions as a psychological buffer against stress and promotes resilience (Schunk and DiBenedetto, 2021). Furthermore, correlations with the external behavioral validators indicated that individuals with higher PFPA scores (greater fear perception) were more likely to report protective behaviors such as mask-wearing, stockpiling, and avoiding social activities. Although the effect sizes were modest, all associations were significant, providing support for the ecological validity of the PFPA.

Compared to other scales, the PFPA distinguishes cognitive dimensions of fear from emotional ones. For example, while the FCV-19S and CAS remain valuable for screening distress and clinical anxiety, the PFPA is more suitable for public health applications, such as evaluating community readiness, designing risk communication strategies, and tailoring interventions to enhance coping. Integrating PFPA assessments into surveillance systems could help policymakers anticipate public reactions to emerging health threats and deploy targeted education campaigns.

Previous studies have developed scales for fear, such as FQ and FCV-19S. FQ primarily focuses on the subjective experience of fear and avoidance behaviors associated with specific phobias (Arrindell and Emmelkamp, 1984). FCV-19S is specifically designed to measure fear related to the COVID-19 pandemic and primarily evaluates the emotional aspect of fear in response to the pandemic threat (Ahorsu et al., 2020). FCV-19S was further verified to have significant association with psychometric characteristics, such as anxiety, stress and depression (Bitan et al., 2020). Compared with existing scales, The PFPA fills a methodological gap by integrating both cognitive and behavioral aspects of fear. Individuals’ perception of fear can be reflected on their self-efficacy, perceived susceptibility, and perceived threat, which explain why and how they experience fear. Moreover, based on the scores of different subscales, insights can be gained by analyzing how different fear perceptions affect behaviors.

Importantly, although established scales such as the CAS capture pandemic-related anxiety effectively, they conceptualize fear primarily as a set of emotional or symptomatic responses (e.g., physiological arousal, worry, panic) (Lieven, 2023). By contrast, the PFPA emphasizes the cognitive and appraisal components of fear (self-efficacy, susceptibility, threat perception) and their link with concrete behaviors. In this sense, PFPA and CAS are complementary: CAS reflects the emotional intensity of pandemic fear, while PFPA situates fear within the framework of health-protective cognition and action. Future studies should correlate the PFPA with the CAS to establish convergent validity and clarify its position among fear assessment tools.

## 5 Conclusion

In conclusion, the PFPA provides a theoretically grounded, psychometrically robust instrument for capturing pandemic fear as a multidimensional construct. Unlike existing scales that focus primarily on emotional symptoms, the PFPA highlights cognitive appraisals of susceptibility, perceived threat, and self-efficacy, and links these constructs with adaptive behaviors. This dual focus allows for richer understanding of how individuals perceive and respond to health crises.

The PFPA was validated using data collected during the sudden and unprecedented wave of COVID-19 infections in China in December 2022. These data capture a unique moment of acute collective fear, making the resulting scale an especially valuable tool for studying psychological responses under real-world crisis conditions. Beyond documenting reactions to COVID-19, the PFPA’s innovative integration of cognition, fear, and behavior provides a flexible framework that can be adapted to future epidemics or other sudden public health threats. Practically, the PFPA can be used in evaluation (monitoring population-level fear and adaptability), intervention (designing targeted campaigns to strengthen self-efficacy and correct misperceptions), and public health strategies (informing preparedness planning and crisis response).

## 6 Limitation and future study

### 6.1 Psychometric limitations

The PFPA showed a relatively low Person Separation Index (PSI = 1.25), suggesting limited sensitivity in distinguishing individuals with different levels of fear. Such findings are common in short scales during early validation stages. In our study, in the context of China’s abrupt relaxation of COVID-19 restrictions in December 2022, participants exhibited high homogeneity in fear responses, potentially reducing response variance. This situational specificity suggests that the PFPA effectively captured collective fear reactions during a crisis, rather than indicating a design flaw. Moreover, internal consistency concerns emerged in the self-efficacy and Perceived susceptibility, which showed weaker fit in Rasch and ICC analyses. It should also be noted that short item subscales typically depress Cronbach’s *α* because reliability coefficients are sensitive to the number of items, even when the items are conceptually appropriate. At last, although the overall internal consistency of the PFPA reached the commonly recommended threshold (α = 0.700) in the final sample, one subscale (perceived susceptibility) showed a Cronbach’s α slightly below 0.70 (α = 0.688). This is consistent with prior psychometric guidance that values between 0.60 and 0.70 are acceptable in early-stage validation, particularly for short scales with few items. Nevertheless, future studies should consider adding or refining items to enhance the reliability of this dimension.

### 6.2 Convergent validity limitations

The PFPA was not directly compared against widely validated pandemic-related fear scales such as the FCV-19S or the CAS. This limits the ability to situate PFPA within the broader measurement landscape. To partially address this, we correlated PFPA with affect-related items adapted from the STAID, which showed expected negative associations, as well as with behavioral items reflecting protective actions, which showed positive associations. While these analyses provided preliminary convergent and external validity evidence, future research should directly examine correlations with FCV-19S, CAS, and related measures in different populations.

### 6.3 Sample composition limitation

The study sample was skewed toward younger adults, reflecting the online convenience recruitment strategy. This demographic imbalance limits generalizability, particularly to older populations who may perceive and respond to pandemic fear differently. Although this sampling strategy was chosen to capture real-time data immediately following the sudden lifting of COVID-19 restrictions, future work should test the PFPA in more representative and age-diverse samples.

### 6.4 Cultural and contextual limitation

The PFPA was developed in the unique sociocultural context of China during the immediate post-lockdown period in December 2022. While this setting provides rare and valuable insights, the findings may not generalize across different cultural contexts. Future studies should adapt and validate the PFPA cross-culturally, testing measurement invariance across diverse populations (e.g., Western countries, Southeast Asia, Africa), where health literacy, risk perception, and pandemic experiences differ.

### 6.5 Design constraints due to emergency conditions

Certain methodological choices—such as limited cognitive pre-testing and reliance on convenience sampling—were constrained by the urgency of the situation. Nevertheless, these data offer an invaluable snapshot of fear and adaptability during a critical transition period. Future studies under more controlled conditions can refine the PFPA psychometric robustness and broaden its practical applications.

## Data Availability

The datasets presented in this study can be found in online repositories. The names of the repository/repositories and accession number(s) can be found below: https://doi.org/10.57760/sciencedb.08884.

## Glossary

PFPA: Pandemic Fear Perception and Adaptability Scale
FSS-III: Fear survey schedule
FQ: Fear Questionnaire
FCV-19S: Fear of COVID-19 Scale
CAS: Coronavirus Anxiety Scale
STAID: State–Trait Anxiety Inventory derived
HBM: Health Belief Model
PMT: Protection Motivation Theory
CTT: Classical Test Theory
AVE: Average variance extracted
DIF: Differential item functioning
MNSQ: Infit and outfit mean square
ICC: Item characteristic curve
CMIN: Chi-Square Minimum Discrepancy
CFI: Comparative Fit Index
GFI: Goodness of Fit Index
AGFI: Adjusted Goodness of Fit Index
TLI: Tucker-Lewis Index
RMSEA: Root Mean Square Error of Approximation
SEM: Standard Error of Measurement
RMR: Root Mean Square Residual
