# Supplementary material for: Development and validation of the pandemic fear perception and adaptability scale
Source: Front Psychol. 2025 Oct 14;16:1661940. doi: 10.3389/fpsyg.2025.1661940 (PMC12558817; doi:10.3389/fpsyg.2025.1661940)
Supplement: Supplementary file 1 [file Data_Sheet_1.pdf]

The supplementary materials illustrate the items in Pandemic Fear Perception and Adaptability Scale (PFPA), as well as the external validators and the convergent validity.

1.1 Items in Pandemic Fear Perception and Adaptability Scale

Table1 shows the detailed information of items and subscales of PFPA. All items are rated on a 7-point Likert scale (1 = totally disagree, 7 = strongly agree). Participants were required to answer the external behavioral validators before completing the Self-Efficacy subscale (Items 1 and 2). Specifically, participants were first asked whether they had engaged in these behaviors (binary yes/no). Then, the self-efficacy subscale assessed their perceived ability to perform these same behaviors and the belief that performing them would be effective.

Table 1 Pandemic Fear Perception and Adaptability Scale

Item Number	Item Wording	Response Scale	Subscales
Item 1	I believe I am able to perform the protective behaviors mentioned above to prevent or cope with COVID-19.	1–7 Likert	Self-efficacy
Item 2	Doing the above actions can help avoid the pandemic (COVID-19) or recover better if I contract the pandemic (COVID-19).	1–7 Likert	
Item 3	I am at risk of getting infected by the pandemic (COVID-19).	1–7 Likert	Perceived Susceptibility
Item 4	I might get infected by the pandemic (COVID-19).	1–7 Likert	
Item 5	I might have been infected with COVID-19.’	1–7 Likert	
Item 6	The pandemic (COVID-19) is very harmful.	1–7 Likert	Perceived Threat
Item 7	The pandemic (COVID-19) is a serious threat to us.	1–7 Likert	

1.2 External behavioral validators

These three questions are binary, with answer yes or no.

‘1. Have you tried to avoid going out and other social activities due to the pandemic (COVID-19)?’

‘2. Have you attempted to purchase and stock up on medications or other items related to preventing or treating the pandemic (COVID-19)?’

‘3. Do you always wear medical masks when going out, even N95 masks? Have you increased the frequency and duration of handwashing?’

Note: the answer yes=1, no=2

2. Six affect-related items adapted from the State-Trait Anxiety Inventory

There are six items assessed feelings of calmness, security, peacefulness, happiness, and relaxation, as well as one item reflecting fearfulness. Most items were positively worded, representing the conceptual opposite of fear. Responses were recorded on a 4-point Likert scale (1 = totally disagree, 4 = strongly agree). This is considered as the convergent validity in study.

Table 2 six State-Trait Anxiety Inventory derived items

Item Number	Item Wording	Response Scale
Item 1	I feel very peaceful	1–4 Likert
Item 2	I feel very secure	1–4 Likert
Item 3*	I am very fearful	1–4 Likert
Item 4	I feel very calm	1–4 Likert
Item 5	I feel very happy	1–4 Likert
Item 6	I feel very relaxed	1–4 Likert

* Item 3 is a contradictory meaning question and it needs to reversely score
